# Social Media and Cyber-Bullying in Autistic Adults

**DOI:** 10.1007/s10803-021-05361-6

**Published:** 2021-11-19

**Authors:** Paraskevi Triantafyllopoulou, Charlotte Clark-Hughes, Peter E. Langdon

**Affiliations:** 1 Tizard Centre, University of Kent, Cornwallis North East, Canterbury, CT2 7NF Kent England; 2 Centre for Educational Development, Appraisal, and Research (CEDAR) and Centre for Mental Health and Wellbeing Research, University of Warwick, Coventry, CV4 7AL England; 3 Rainbow Unit, Coventry and Warwickshire Partnership NHS Trust, Birmingham, B37 5RY England; 4 Herefordshire and Worcestershire Health and Care NHS Trust, Worcester, WR5 1JR England

**Keywords:** Autism, Social media, Cyber-bullying victimisation, Cyber-aggression, Internet

## Abstract

Social media can lead to rejection, cyber-bullying victimisation, and cyber-aggression, and these experiences are not fully understood as experienced by autistic adults. To investigate this, 78 autistic adults completed self-report measures of social media use, cyber-bullying victimisation, cyber-aggression, and self-esteem. High levels of social media use were found to be associated with an increased risk of cyber-victimisation; whereas self-esteem was positively correlated with feelings of belonging to an online community and negatively correlated with feelings of being ignored on social network sites and chat rooms. Future studies are needed to further investigate the experience of cyber-bullying victimisation of autistic adults.

**Supplementary Information:**

The online version contains supplementary material available at 10.1007/s10803-021-05361-6.

## Introduction

Social media has added another dimension to our communication, interaction, and connection with others, and is well integrated into our lives. Social media can broadly be defined as a group of emerging online media which facilitate social interaction; these platforms encompass internet applications such as social networking websites and online forums, as well as virtual gaming (O’Keeffe & Clarke-Pearson, 2011). The accessibility, availability, and immediacy of social media (i.e., instant messaging), have made them extremely popular, and the method of choice for the promotion of social engagement (Mazurek & Engelhardt, 2013). Statista (Tankovska, 2021) reported 93% of adults in the UK to have had their own social network profile in 2019. It has been suggested that social media use among young adults often provide enhanced social engagement and functioning (Gross et al., 2002). Online communication has also been suggested to help maintain and strengthen relationships (Reich et al., 2012) resulting in closer friendships and increased friendship quality in adolescents (Blais et al., 2008).

We are stating the obvious by saying that some autistic people use social media for entertainment and to socially connect with others and communicate (Mazurek, 2013). Nevertheless, autism is construed as a lifelong neurodevelopmental condition characterised by deficits in social interaction and communication alongside restricted, repetitive, or stereotyped patterns of behaviours, interests, and activities (American Psychiatric Association, 2013) affecting about 1% of the population (Russell et al., 2015). Face-to-face interactions are sometimes challenging for autistic individuals due to problems with recognising and processing facial expressions (Rump et al., 2009; Smith et al., 2010). Therefore, social media have been reported as one particularly attractive alternative avenue of communication to help establish social relationships, enhance friendships and reduce the feelings of loneliness in autistic individuals (Blume et al., 2001; Mazurek et al., 2012; Kraut et al., 2002; Shaw & Gant, 2002; Valkenburg & Peter, 2007).

Despite its positive aspects, social media use also comes with certain risks, which may be greater for those who have difficulties understanding and responding to social cues (Kowalski & Fedina, 2011). It has been argued that social media use may lead to victimisation rather than positive social interaction (DiMaggio et al., 2001). Other risks include social rejection, cyber-bullying, depression, and other negative consequences related to general well-being (O’Keefee & Clarke-Pearson, 2011; Valkenburg & Peter, 2007). The use of technology to advertently harm, annoy or defame others is called ‘cyber-bullying’ (Kowalski & Limber, 2013). Cyberbullying is often defined as a form of intentional aggressive behaviour which is then repeated over a period of time. This is done in a deliberate manner to cause significant discomfort or harm (Agatston et al., 2007) and is carried out through the use of electronic means such as: e-mail, social media, images, digital messages, online gaming etc. (Kowalski et al., 2014; Olweus, 1993).

The prevalence of cyber-bullying victimisation is estimated between 37 and 70% for the general population (Microsoft, 2012; Cramer, 2018). Past research has indicated that individuals with specific types of disabilities are more likely to be victimised in comparison to others, with specific characteristics of their disability enhancing their vulnerability and risk of being targeted (Twyman et al., 2010). Cyber-bullying victimization has been reported for autistic adolescents in China (15%; Hu et al., 2019), Spain (64.4%; Iglesias et al., 2019) and Canada (30.4%; Holfeld et al., 2019), with the differences in prevalence explained possibly by sample differences between the studies. Low levels of cyber-aggression enacted by autistic adolescents has been reported (Hu et al., 2019; Iglesias et al., 2019; Holfeld et al., 2019) with other studies reporting low involvement of autistic adults in cybercrime (Ledingham & Mills, 2015; Payne et al., 2019).

Authors of previous studies have linked bullying with adverse psychological effects such as depression, low self-esteem (Didden et al., 2009; Kowalski & Limber, 2013) and anxiety (Holfeld et al., 2019), with cyber-bullying being associated with behavioural, mental health (Holfeld & Mishna, 2019; Kowalski et al., 2014), and self-esteem (Cenat et al., 2014; Perren et al., 2010) problems. Depression is associated with cybervictimization in adolescents with intellectual and developmental disabilities (Wright, 2017) and excessive social media use is associated with low self-esteem (Kalpidou et al., 2011; Mehdizadeh, 2010), even though some studies have reported the exact opposite in typically developed adolescents (Valkenburg et al., 2005).

### Current Study

Considering the lack of previous research studies on the topic, we completed an online survey to further understand the nature of social media use, cyber-bullying victimisation and cyberaggression amongst autistic adults and whether this is related to their wellbeing. The specific aims of this study were: (i) to investigate social media use in autistic adults, (ii) to examine the occurrence of cyber-bullying victimisation and cyber-aggression in this population, (iii) to examine the relationship between self-esteem and social media use, including time spent online, and cyber-bullying victimization and cyber-aggression.

## Method

### Participants

Data collection took place in 2017 and 2018. In total, 81 individuals opened the online link to the survey, and 78 autistic adults completed at least 70% of the questionnaires. Twenty-nine participants were male (37.2%), 43 were female (55.1%) and 6 preferred not to state their gender (7.7%). Participant ages ranged from 18 to 59 years (*M* = 29.3, SD = 9.7) and most were white European (79.2%). All participants confirmed that they had a diagnosis of autism, and all reported to have access to a personal computer, a laptop, or a smartphone. Just under one half (48.7%) were currently in education, 16.7% had a postgraduate degree in higher education, 29.5% had an undergraduate degree, and 38.5% had A level (or equivalent) qualifications. The cognitive ability and autism severity of participants was not assessed.

### Procedure

The study was advertised online in relevant autism specific sites and forums. Participation was voluntary and all participants had to provide informed consent prior to completing the questionnaires which were presented using Qualtrics. A hyperlink was made available in the study advertisement that directed potential participants to an online information sheet and consent form. Participants were required to accept participation and give their consent before accessing the online questionnaires. Their identity could be kept anonymous if they did not wish to provide their name. The questionnaires took approximately 15 min to complete. Ethical approval was gained from the University of Kent prior to the start of the study. Additional information regarding the participants’ age, gender, ethnic background, educational background, diagnosis, as well as access to electronic devices, was also collected.

#### Questionnaires

##### Adapted Facebook Intensity Scale (FIS)

An adapted version of the FIS (Ellison et al., 2007) was used to measure the participants’ social media usage and attitudes, looking at the extent to which participants were emotionally connected to social media, and the extent to which they were integrated in their social activities. Participants were presented with 8 self-report individual item statements (e.g., “*Social media is part of my everyday activity”*) and were asked to respond to each statement using a 5-point Likert-scale, ranging from 1 = *Strongly Disagree* to 5 = *Strongly Agree*. The original FIS was developed to measure Facebook usage, however, as the current study was interested in the use of social media in general, all Facebook references within the FIS were replaced by ‘social media’. This was the only change made hence the psychometric properties of the original scale were not compromised. The scoring of the adapted version of FIS followed the scoring of the original scale. Therefore, a mean of each item scale was calculated, and the total score achievable ranged from 6 to 30, following the original FIS. The sum of scores indicated participants’ social media usage, with higher scores representing greater use of social media.

##### European Cyber-Bullying Intervention Project Questionnaire (ECIPQ)

The ECIPQ (Brighi et al., 2012) was used to assess cyber-bullying victimisation and cyber-aggressive behaviour. Participants were presented with 22 self-report individual item statements, split equally between items that examine cyber-bullying victimisation, and cyber-aggression. They were asked to indicate how often they had experienced or taken part in specific behaviours, in the past 2 or 3 months. For example, “*Someone threatened me through texts or online messages*” and “*I threatened someone through texts or online messages*”. Participants answered on a 5-point Likert scale, ranging from 0 = *never,* 1 = *once or twice,* 2 = *once per month,* 3 = *once per week,* 4 = *more than once per week*. The ECIPQ was scored based on the scoring criteria outlined by Del Rey et al., (2015), and total scores were calculated separately for cyber-aggression and cyber-bullying victimization.

##### Rosenberg Self-Esteem Scale (RSES)

The RSES was used to assess participants’ level of self-esteem (Rosenberg, 1965). This measure is comprised 10 individual self-report items (for example, ‘*On the whole I am satisfied with myself’*) rated on a 4-point Likert scale, ranging from 0 = *Strongly agree* to 4 = *Strongly disagree*. Participants were asked to decide the extent to which they agreed with each statement. The RSES has been extensively used with autistic individuals in past research (e.g., Mazurek, 2014; Hillier et al., 2018; Gordon et al., 2015), has test–retest reliability that ranges from 0.82 to 0.88, and internal consistency that ranges from 0.77 to 0.88 (Blascovich & Tomaka, 1993; Rosenberg, 1986; Schmitt & Allik, 2005). Participant total scores were calculated, and scores can range from 0 to 30. Higher scores indicate greater levels of self-esteem.

### Analysis

For the first aim, to investigate social media use in autistic adults, descriptive statistics were calculated and summarised. For the second aim, to examine the occurrence of cyber-bullying victimisation and cyber-aggression in autistic adults, descriptive statistics were also calculated and summarised. The Wilcoxon Sign-Ranked test was then used to compare differences between those with a history of cyber-bullying victimisation and cyber-aggression. For the third aim, initially, a series of Spearman’s rho correlation coefficients were used to examine the relationships between self-esteem (RSES) and social media (adapted-FIS) use, as well as cyber-bullying victimisation and cyber-aggression data (ECIPQ). Finally, linear regression was used to examine the relationship between either experience of cyber-bullying victimisation or cyber-aggression and age, gender, educational qualifications, time spent online, and the number of friends on social media.

## Results

### Social Media Use (Adapted FIS)

All participants reported to have access to a device that could connect to the internet, and all participants reported using social media (100%); 27.3% of participants said that they spent between 1 and 2 h per day on social media (n = 21), while 16.9% of participants indicated that they spent between 2 and 3 h per day on social media (n = 13); 16.9% indicated they spent 30–59 min on social media. Only 5.2% of participants indicated that they used social media more than 5 h per day (n = 4). The majority of the participants also reported to have more than 200 friends on their social media accounts (28.6%, n = 22) with 15.6% of participants having 10 friends or less (n = 12).

The findings from the adapted FIS (Table 1), which measured social media usage and attitudes, and the extent that social media was part of everyday life suggested that social media was a big part of everyday life and everyday routine, with 85.7% of participants agreeing it was part of everyday activity, and 77.9% agreeing that it was part of their daily routine.

**Table 1:** Descriptive statistics for adapted FIS items of the questionnaire

Adapted FIS items	M (SD) N = 77	Agreement-frequencies (%)
Social media is part of my everyday activity	4.25 (1.24)	66 (85.7%)
I am proud to tell people I am on social media	3.01 (1.17)	22 (28.6%)
Social media has become part of my daily routine	4.16 (1.26)	60 (77.9%)
I feel out of touch when I have not logged into social media	3.32 (1.44)	40 (52%)
I feel that I am part of a social media community	3.27 (1.29)	37 (48.1%)
I would be sorry if social media shut down	3.40 (1.47)	42 (54.6%)
Number of friends on social media	4.14 (2.49)	–
Time spent on social media	4.00 (1.65)	–
Adapted FIS total	21.41 (5.99)	–

### Experiences of Cyber-bullying Victimisation and Cyber-aggression (ECIPQ)

Participants’ experiences of cyber-bullying victimisation and cyber-aggression was measured using the ECIPQ. Thirty one percent reported that during the past 2–3 months they had experienced cyber-bullying victimisation (n = 23), 2.7% reported to have engaged in cyber-aggressive behaviours (n = 2), and 8% reported to have experienced both cyber-bullying victimisation and acted as cyber-aggressors (n = 6). In contrast to this, 58.7% had no experience of either cyber-bullying victimisation or cyber-aggression. Twenty-four percent (n = 19) of the participants stated that they were excluded or ignored by others in a social networking site or internet chat room, 23% (n = 18) stated that someone said nasty things about them to others online, and 22% (n = 17) stated that someone said nasty things to them or called them names using online messages. The mean total cyber-victimisation score was 4.88 (SD = 5.75), and the mean total cyber-aggression score was 1.61 (SD = 3.33). Overall, participants reported experiencing significantly higher cyber-bullying victimisation relative to engaging in cyber-aggression (*z* = 5.51, *p* < 0.001). Table 2, below, shows a breakdown of the reported frequencies for each of the ECIPQ statements, as well as the total ECIPQ scores for both cyber-bullying victimisation, and cyber-aggression.

**Table 2:** Descriptive statistics for ECIPQ items of the questionnaire

	Never [% (n)]	Once or twice [% (n)]	Once per month [% (n)]	Once per week [% (n)]	More than once per week [% (n)]	Mean* (SD) (n = 75)
Cyber-bullying victimisation items						
Someone said nasty things to me or called me names using texts or online messages	44% (33)	33% (25)	13% (10)	4% (3)	5% (4)	0.93 (1.11)
Someone said nasty things about me to others either online or through text messages	51% (38)	25% (19)	9% (7)	5% (4)	9% (7)	0.97 (1.29)
Someone threatened me through texts or online messages	63% (47)	28% (21)	5% (4)	4% (3)	–	0.51 (0.78)
Someone hacked into my account and stole personal information	89% (67)	9% (7)	–	–	1% (1)	0.15 (0.54)
Someone hacked into my account and pretended to be me	95% (71)	4% (3)	1% (1)	–	–	0.07 (0.3)
Someone created a fake account, pretending to be me	91% (68)	7% (5)	1% (1)	1% (1)	–	0.13 (0.47)
Someone posted personal information about me online	72% (54)	20% (15)	5% (4)	1% (1)	1% (1)	0.4 (0.77)
Someone posted embarrassing videos or pictures of me online	80% (60)	16% (12)	4% (3)	–	–	0.24 (0.52)
Someone altered pictures or videos of me that I had posted online	89% (66)	11% (8)	–	–	–	0.11 (0.31)
I was excluded or ignored by others in a social networking site or internet chat room	53% (40)	21% (16)	4% (3)	8% (6)	13% (10)	1.07 (1.45)
Someone spread rumours about me on the internet	71% (53)	16% (12)	8% (6)	3% (2)	3% (2)	0.51 (0.95)
*Total Cyber-victimisation*						4.88 (5.75)
Cyber-aggression items						
I said nasty things to someone or called them names using texts or online messages	79% (59)	15% (11)	1% (1)	3% (2)	3% (2)	0.36 (0.86)
I said nasty things about someone to other people either online or through text messages	72% (54)	17% (13)	8% (6)	1% (1)	1% (1)	0.43 (0.81)
I threatened someone through texts or online messages	92% (69)	7% (5)	1% (1)	–	–	0.09 (0.34)
I hacked into someone’s account and stole personal information	97% (73)	3% (2)	–	–	–	0.03 (0.16)
I hacked into someone’s account and pretended to be them	99% (74)	–	–	1% (1)	–	0.04 (0.35)
I created a fake account, pretending to be someone else	92% (69)	7% (5)	1% (1)	–	–	0.09 (0.34)
I posted personal information about someone online	99% (74)	–	1% (1)	–	–	0.03 (0.23)
I posted embarrassing videos or pictures of someone online	93% (70)	5% (4)	1% (1)	–	–	0.08 (0.32)
I altered pictures or videos of another person that had been posted online	91% (68)	8% (6)	1% (1)	–	–	0.11 (0.35)
I excluded or ignored someone in a social networking site or internet chat room	76% (57)	21% (16)	1% (1)	–	1% (1)	0.29 (0.63)
I spread rumours about someone on the internet	95% (71)	4% (3)	1% (1)	–	–	0.07 (0.30)
*Total cyber-aggression*						1.61 (3.33)

*0 = never, 1 = once or twice, 2 = once per month, 3 = once per week, 4 = more than once per week

### Relationship Between Self-Esteem, Social Media Use, Cyber-bullying Victimisation and Cyber-Aggression

Participants scored an average of 15 out of a possible 30 on the RSES, which measured self-esteem, with higher scores indicating greater levels of self-esteem (*M* = 15(6.24), n = 78). Self-esteem was positively related to the number online friends, r(77) = 0.27, p = 0.02, and seeing oneself as part of an online community, r(77) = 0.29, p = 0.01, as measured by the FIS. There was a negative relationship between self-esteem and cyber-bullying victimisation (ECIPQ), specifically with respect to how often someone else posted personal information about the participants online r(75) = − 0.25, p = 0.03, as well as the amount of times participants were excluded or ignored by others on social media, r(75) = − 0.29, p = 0.01.

### Relationship Between Time Spent on Social Media, Self-Esteem and Experiences of Cyber-Bullying Victimisation and Cyber-Aggression

#### Model 1: Cyber-Bullying Victimisation

A multiple linear regression analysis was carried out in order to model the linear relationship between the time spent on social media, experiences of cyber-bullying victimisation, cyber-aggression, and self-esteem. Model 1 predicted cyber-bullying victimisation, with a significant regression equation (F(5, 62) = 5.796, p < 0.001) and a regression coefficient R = 0.564, suggesting a strong relationship. The adjusted R^2^ = 0.319, meaning that 31.9% of the variance in the cyber-victimisation, was explained by the five predictor variables. See Table 3 for more information about the relationship between time spent on social media and experiences of cyber-bullying victimisation, cyber-aggression, and self-esteem.

**Table 3:** Model 1: regression for time spent on social media and cyber-bullying victimisation for autistic adults

	Unstandardized Coefficients		Standardized Coefficients	t	Sig
	B	SE	Beta		
*Model 1*					
Cyber-bullying Victimisation (ECIPQ)	3.097	3.258		.951	.345
Age in years	.133	.066	.220	2.010	.049
Male	− 2.812	1.306	− .237	− 2.153	.035
Educational qualifications	.856	.559	.170	1.532	.131
Time spent on social media (adapted FIS)	5.228	1.308	.430	3.998	.000
Social media friends (adapted FIS)	− 1.458	1.287	− .125	− 1.133	.262

Out of the five, there were three statistically significant predictors; time spent on social media (β_time_ = 0.430; t = 3.998; p < 0.001), gender (β_gender_ = − 0.237; t = − 2.153; p = 0.03) and age (β_age_ = 0.220; t = 2.010; p = 0.04). The educational qualifications and the number of social media friends were not significant predictors of cyber-victimisation.

#### Model 2: Cyber-Aggression

Model 2 predicted cyber-aggression. However, the regression equation was not significant (F(5, 63) = 0.166, p > 0.05) and the coefficient was R = 0.114. Only 1.3% of the variance in the cyber-aggression was explained by the five predictor variables (R^2^ = 0.013). From the independent variable regression coefficients, none of the predictors were statistically significant. In other words, while the time spent on social media was a significant predictor of cyber-bullying victimisation, it was, however, not a significant predictor for cyber-aggression. Table 4 provides additional information about the relationship between time spent on social media and experiences of cyber-bullying victimisation, cyber-aggression, and self-esteem**.**

**Table 4:** Model 2: regression for time spent on social media, cyber-bullying victimisation and cyber-aggression for autistic adults

	Unstandardized coefficients		Standardized coefficients	t	Sig
	B	SE	Beta		
*Model 2*					
Cyber-aggression (ECIPQ)	3.339	2.317		1.441	.154
Age in years	− .010	.047	− .028	− .210	.834
Male	− .674	.925	− .096	− .728	.469
Educational qualifications	− .101	.397	− .034	− .255	.800
Time spent on social media (adapted FIS)	− .044	.926	− .006	− .048	.962
Social media friends (adapted FIS)	.041	.911	.006	.045	.964

## Discussion

Until now, no other study has explored the relationship between cyber-bullying victimisation, cyber-aggression and social media use in autistic adults. The primary aim of the study was to investigate social media use and examine the occurrence of cyber-bullying victimisation and cyber-aggression in autistic adults. Social media was found to be a big part of autistic adults’ everyday life. In the current study, participants reported social media to be part of their daily routine, feeling ‘out of touch’ when they had not logged in their social media. This is comparable to previous studies also looking at social media use for autistic adults (Mazurek, 2013). As Facebook is no longer the dominant online platform (Lenhart et al., 2015), the current study looked at social media in general. Autistic adults made use of social media more relative to Ellison et al. (2007) who investigated Facebook use in undergraduate university students. Past research has also shown a positive relationship between autistic traits and compulsive internet use in young adults (Shane-Simpson et al., 2016), which might have also contributed to the observed elevated rates.

Participants also reported higher rates of cyber-bullying victimisation than cyber-aggression in the current study. The prevalence rates of cyber-bullying victimisation reported here (37%) is in agreement with prevalence data from the general population (37–70%; Microsoft, 2012; Cramer, 2018), and with prevalence rates reported for autistic adolescents in Canada (30.4%; Holfeld et al., 2019). Participants in the current study seem to score higher in cyber-bullying victimisation, when compared to typically developed adolescents and young adults aged 11–23, from 6 different countries (Del Rey et al, 2015). Cyber-aggression rates were low in the current study, and this is also in agreement with data collected from autistic adolescents (Hu et al., 2019; Iglesias et al., 2019; Holfeld et al., 2019).

Whilst exploring experiences of cyber-bullying victimisation and cyber-aggression in autistic adults, as well as social media intensity, the current study also looked further into how these factors may have an association with autistic adults’ self-esteem. Previous research has highlighted the negative impact of traditional bullying on individuals’ well-being by increasing their anxiety, depression and fear (Espelage & Swearer, 2003). The negative psychological effects of cyber-bullying victimisation on self-esteem (Extremera et al., 2018; Palermiti et al., 2017; Patchin & Hinduja, 2010) and excessive social media use on self-esteem (Kalpidou et al., 2011; Mehdizadeh, 2010), has been well documented in the general population. However, no other study has investigated the relationship between self-esteem, cyber-bullying victimisation, cyber-aggression, and social media use in autistic adults. Self-esteem rates were found to be highly correlated with feelings of belonging in an online community and negatively correlated with feelings of being ignored on social network sites and chat rooms. The feeling of belonging in an online community had a positive effect on participants, promoting feelings of pride and usefulness, whereas being ignored on social media elevated feelings of worthlessness and negativity.

The current study also found that high levels of social media use were associated with an increased risk of cyber-bullying victimisation amongst autistic adults. Adults who spent more than 2 h per day on social media, scored higher on cyber-bullying victimisation when compared to those who spent less than 2 h per day on social media. This is in line with previous research on autistic adolescents with Attention Deficit Hyperactivity Disorder (Kowalski & Fedina, 2011). Mazurek (2013), in their study also looking at social media use in autistic adults, reported that the majority of their participants used social media to keep in touch with friends and family. Keeping in mind the particular challenges autistic individuals may come across with face-to-face interactions, social media are an attractive alternative mean of communication. Therefore, the associations between cyber-bullying victimisation and social media use highlighted by the current study, are particularly alarming for the autistic population.

### Limitations

Although this is the first study looking at the relationship between cyber-bullying victimisation, cyber-aggression, self-esteem and social media use in autistic adults, there are some limitations that should be noted. The generalisation of the current study’s findings should be treated with caution due to the low number of participants involved in the study. Participants were volunteers who might have responded to the call for this study due to their high use of social media and experiences of cyber-bullying victimisation. All measures administered to participants were self-reported measures. Although this could be viewed as a methodological strength in comparison to previous studies (Didden et al., 2009; Kowalski & Fedina, 2011), it also applied some limitations to the study. The data collected represents participants’ own perceptions and memories of cyber-bullying victimisation, cyber-aggression, and social media use. The majority of the participants who responded to the study’s call were females, while typically the gender ratio for autism is 3:1 male to female (Loomes et al., 2017). However, no gender differences were highlighted in any of the variables investigated. The participants’ cognitive ability and severity of autism was not assessed in the current study, however from the educational level of the majority of the participants, it can be assumed that participants were of average/ high cognitive ability. Additional variables such as type of devices, platforms used, as well as the positive use of social media and risks taken should be included in future research. Finally, it is important to highlight that the data collection of this study took place in 2017 and 2018 and the online experiences of autistic adults may have changed since then, especially with the COVID-19 pandemic. However, the current study has created an invaluable source of comparable data to support further studies looking at how autistic adults’ experiences may have changed since the pandemic.

## Conclusion

Even though social media were found to be a big part of autistic adults’ everyday life, the results also showed that more adults experienced cyber-bullying victimisation than cyber-aggression. Self-esteem rates were found to be highly correlated with feelings of belonging in an online community and negatively correlated with feelings of being ignored on social network sites and chat rooms, whereas high levels of social media use were associated with an increased risk of cyber-bullying victimisation amongst autistic adults. These results have implications for public awareness, policy makers, and clinicians working in adult health and social services. Future research is required to further investigate social media use and cyber-bullying victimisation in autistic adults, as well as its possible association with self-esteem. There is also a clear need for intervention and increase of awareness around the risks and occurrences of cyber-bullying in this population.

## Supplementary Information

Below is the link to the electronic supplementary material.

Electronic supplementary material 1 (SAV 88 kb)
